# Psychometric evaluation of the reliability and validity of the literacy of suicide scale among Chinese nurses

**DOI:** 10.3389/fpsyg.2025.1480813

**Published:** 2025-06-02

**Authors:** Zheshu Xie, Juanjuan Lin, Ying Fan, Feirong Tan, Yumei Zhou, Xing Liu

**Affiliations:** ^1^Department of Nursing, Xiangyang No. 1 People's Hospital, Hubei University of Medicine, Xiangyang, China; ^2^School of Nursing, Zhejiang Chinese Medical University, Hangzhou, China; ^3^Department of Pediatrics, Xiangyang No. 1 People's Hospital Affiliated with Hubei University of Medicine, Xiangyang, China

**Keywords:** nurse, suicide literacy, factor analysis, reliability, validity

## Abstract

**Objective:**

This study aimed to Sinicize the Literacy of Suicide Scale (LOSS) and to test the reliability and validity of the LOSS with Chinese nurses.

**Methods:**

After authorization was obtained from the original author, the LOSS was translated into Chinese and translated back into English in accordance with Brislin’s translation principle. Eight experts were invited to evaluate the scale’s content validity, and the Chinese version of the LOSS was obtained. Moreover, the LOSS was used to assess the suicide literacy of 1,000 nurses from Beijing, Hubei, Henan, and Sichuan Provinces in China, and the reliability and validity of the scale were tested.

**Results:**

The Chinese version of the LOSS contains 26 items covering four dimensions: signs of suicide, risk factors for suicide, the cause/nature of suicide, and the treatment/prevention of suicide. Cronbach’s *α* coefficient for the LOSS was 0.933, and Cronbach’s *α* coefficients of the four dimensions were 0.832, 0.893, 0.898, and 0.827. The split-half reliability of the LOSS was 0.818, and the split-half reliabilities of the four dimensions were 0.835, 0.877, 0.890, and 0.819. The test–retest reliability of the LOSS was 0.925, and the test–retest reliabilities of the four dimensions were 0.890, 0.885, 0.892, and 0.904. The item-level content validity index (I-CVI) of the scale was 0.875–1.000, and the scale-level content validity index (S-CVI) was 0.947. Four common factors were extracted via exploratory factor analysis, and the cumulative variance contribution rate was 60.233%. The confirmatory factor analysis results show that the model had a good fit.

**Conclusion:**

The Chinese version of the LOSS has good reliability and validity and is a suitable assessment tool for assessing nurses’ suicide literacy in the Chinese cultural context.

## 1 Introduction

Suicide has become an important public health and mental health problem affecting the health of the global population. According to the World Health Organization, at least 800,000 people die by suicide every year worldwide (Bachmann, 2018). The annual death rate from suicide in China is approximately 8 deaths per 100,000 people (Snowdon and Choi, 2020). Hospitalized patients are at increased risk of suicide because of the suffering caused by their illnesses. A total of 600–800 suicides per 100,000 hospitalized people occur per year, which is nearly 50–72 times higher than the rate in the general population (Gupta et al., 2022). According to reports, the suicide rate of hospitalized patients in general hospitals is approximately 3.26 cases per 100,000 patients (Wan et al., 2020). A study of hospitalized patients in China revealed that nearly 6% had severe suicidal ideation (Huang et al., 2019). The harm caused by inpatient suicide, which can cause patients and their families to experience serious mental trauma and heavy economic burdens, is extensive (Sabrinskas et al., 2022; Viard et al., 2023). Moreover, the deterioration of doctor–patient relationships and medical disputes can easily occur, which places a heavy burden on the social medical service system (Chammas et al., 2022; Gebhardt et al., 2022). In addition, research has shown that medical staff who have experienced inpatient suicide are vulnerable to mental distress, which can negatively affect their work and lives (Senf et al., 2022). In clinical settings, members of the nursing staff serve as frontline caregivers for suicidal patients, providing essential monitoring, support, and intervention (Shih et al., 2023). Nurses’ proximity to patients enhances their capacity to identify suicidal ideation early and to implement preventive interventions (Haddad and Young, 2022). Effective management of suicidal patients requires all nursing staff members, regardless of their clinical specialty, to maintain competency in risk assessment and to possess appropriate intervention resources (Fontão et al., 2018). Upon identifying a patient with suicidal ideation, nurses should employ clinical observation and therapeutic communication techniques to evaluate risk factors and implement appropriate interventions on the basis of the assessed level of danger (Hagen et al., 2017). On the basis of the Nursing Interventions Classification (NIC), the existence and degree of suicide risk can be determined, patients can be asked whether they are planning suicide, the safety of their surroundings can be assessed to prevent them from harming themselves, and a secure environment can be ensured (Muehlenkamp et al., 2023). Therefore, in clinical settings, nurses’ own abilities in suicide prevention and intervention are crucial for preventing and treating suicide among hospitalized patients.

Suicide literacy refers to the extent to which the public understands the causes, risk factors, signs, treatment, and prevention of suicide, and it reflects the level of individual knowledge about suicide (Batterham et al., 2013). The team of Professor Calear coined the term “suicide literacy” in 2012 on the basis of the theory of mental health literacy (Batterham et al., 2013). Suicide literacy has gradually drawn the attention of relevant scholars, and the suicide literacy of teachers, students, community populations, and clinical populations has been researched (Calear et al., 2022; Ludwig et al., 2022; Yamaguchi et al., 2023; Zilinskas and Lesinskienė, 2023). However, few studies have investigated nurses’ suicide literacy. A survey of 348 nurses revealed an overall low suicide literacy score of only 11.08 ± 3.92 points (Karakaya et al., 2023). The evidence suggests that low suicide literacy among nurses predisposes them to stigmatizing patients who attempt suicide and hinders their ability to assess and care for such patients (Shao et al., 2021). In contrast, high suicide literacy among nurses facilitates early recognition of warning signs of suicide in hospitalized patients and the adoption of timely and effective nursing care to save patients’ lives (Boukouvalas et al., 2020). In addition, as nurses’ suicide literacy improves, they are more likely to have positive attitudes toward individuals who attempt suicide, which encourages individuals who attempt suicide to seek help from nurses (Boukouvalas et al., 2020). As the healthcare professionals with the most extended duration of contact with hospitalized patients, nurses are most likely to recognize early signs of suicide in patients and to intervene proactively. Therefore, enhancing nurses’ suicide literacy to prevent suicide in hospitalized patients is crucial.

Before nurses’ suicide literacy can be increased, it is necessary to validate a specific tool for measuring suicide literacy among nurses. In 2021, Calear et al. designed the Literacy of Suicide Scale (LOSS) on the basis of the mental health literacy framework. The LOSS includes 26 items and 4 dimensions, including signs of suicide, risk factors for suicide, the cause/nature of suicide, and the treatment/prevention of suicide (Calear et al., 2022). Currently, the LOSS is applied in many countries and populations and has shown good reliability and validity (Aldalaykeh et al., 2020; Al-Shannaq and Aldalaykeh, 2023; Bekaroğlu et al., 2024; Chan et al., 2014; Han et al., 2017; Jafari et al., 2023). This reality suggests that the LOSS is a credible tool for measuring suicide literacy in different cultural and linguistic contexts. However, the LOSS has not been translated into Chinese, and specific tools for evaluating nurses’ suicide literacy in China are still lacking.

Therefore, this study evaluated the LOSS, Sinicized it, and determined its reliability and validity in the nurse population to provide an excellent assessment tool for studying the level of suicide literacy among Chinese nurses.

## 2 Methods

### 2.1 Research design and research subjects

A multicenter, online cross-sectional survey was conducted in China from January to February 2024. This research was a quantitative study. Eligible nurses were recruited from Beijing, Hubei, Henan, and Sichuan Provinces in China via a convenience sampling method. The inclusion criteria were registered nurses aged 18 years and older who were actively working in a hospital and able to complete an online questionnaire and provide informed consent. There were no exclusion criteria. Considering that the sample size should be 10 to 20 times the number of items in the scale, the sample size was calculated as 260–520 cases. Considering a dropout rate of 20%, the sample size was calculated as 325–650 participants. Ultimately, 1,000 participants were included in the study.

### 2.2 Instruments

#### 2.2.1 Demographic questionnaire for nurses

The general demographic characteristics questionnaire for this study was developed after a systematic literature review and rigorous team discussion (Chan et al., 2014; Fekih-Romdhane et al., 2022). Eight variables were included in the questionnaire: department, age, sex, marital status, educational background, title, position, and employment form.

#### 2.2.2 The literacy of suicide scale

The LOSS, which was developed by Professor Calear’s et al. (2022) team on the basis of the mental health literacy framework, was used in this study. The scale consists of 26 items, which can be divided into four dimensions: signs of suicide (5 items), risk factors for suicide (7 items), the cause/nature of suicide (10 items), and the treatment/prevention of suicide (4 items). Each item on the LOSS is scored in the same way, with a score of 1 for responses of “correct” and a score of 0 for responses of “wrong” or “I do not know.” Items 1, 4, 6, 10, 13, 14, 15, 16, 17, 18, 19, 20, 22, 23, 25, and 26 are reverse scored. The total score of the scale ranges from 0 to 26 points. The higher the score, the greater the level of suicide literacy. Cronbach’s *α* coefficient of the LOSS is 0.87, indicating that the LOSS has good internal consistency (Karakaya et al., 2023).

### 2.3 Procedures

#### 2.3.1 Translation, back-translation, and transcultural adaptation of the LOSS

After authorization was obtained from the original author via email, the LOSS was translated according to the internationally popular Brislin translation principles (Jones et al., 2001). First, two researchers with doctoral degrees in nursing and psychology and with overseas study experience translated the LOSS into Chinese, and they held discussions to develop the Chinese version of the scale. Second, two other researchers who had no access to the original scale (one with a master’s degree in English and the other with a master’s degree in nursing) back-translated the Chinese version of the LOSS into English. In addition, to ensure language conformity, eight experts from universities and tertiary hospitals (2 psychology professors, three chief physicians in a department of psychology, one chief nurse in a department of psychology, and two psychotherapists with senior associate titles) were invited to evaluate the translated scale and propose suggestions for improvement. In accordance with the expert opinions, the final Chinese version of the LOSS was created. Finally, the researchers used a convenience sampling method to select 25 hospital nurses for preliminary investigation, asking the nurses to evaluate the semantic ambiguity of the scale, determine whether the items were easy to understand, and record the total time spent completing the questionnaire. Data from this presurvey were not included in the formal reliability and validity tests.

#### 2.3.2 Data collection procedure

A questionnaire survey was conducted among nurses from four provinces or municipalities in North China, Central China, East China, and Southwest China. The researchers collected data at the target hospitals in each province/municipality during the study period. With the assistance of the directors of the nursing departments in each target hospital, the researchers first explained the research purpose, significance, and precautions regarding the completion of the questionnaire to the head nurses during an online meeting. They then provided the two-dimensional code for the questionnaire. The head nurses then distributed the QR code to the department nurses. The online questionnaire included a demographic questionnaire for nurses and the Chinese version of the LOSS. The China Juanxing app produced the online questionnaire. Each IP address could be used only once to complete the questionnaire. The completion time was no more than 10 min. The descriptions of the research purpose, research significance, and completion method were set as mandatory items, and the questionnaire could be completed only after the participants read the items for 1 min. After questionnaire collection, software was used to export the survey data for data analysis, which avoided errors caused by manual data entry. Ultimately, 1,017 eligible nurses agreed to participate in the study and completed the anonymous questionnaire. The researchers eliminated 17 invalid questionnaires and recovered 1,000 valid questionnaires, for a valid questionnaire rate of 98.33%.

#### 2.3.3 Data analysis procedure

##### 2.3.3.1 Item analysis

The critical ratio (CR) and Pearson’s correlation methods were used for item analysis. (1) The CR method involves ranking the total scale scores from the highest to the lowest, with the top 27% included in the high-score group and the bottom 27% included in the low-score group. Independent sample *t*-tests were used to compare the differences in scores between the two groups. If the CR of an item was greater than 3.0 and the *p*-value was less than 0.05, the item was considered to have good differentiation (Henttonen et al., 2022). (2) The Point–Biserial correlation method involved calculating the correlation coefficient between each item’s score and the scale’s total score. If the correlation coefficient was less than 0.3, the item was deleted (Warholak et al., 2023).

##### 2.3.3.2 Reliability analysis

Cronbach’s *α* coefficient and split-half reliability were used to evaluate the internal consistency of the Chinese version of the LOSS. The time stability of the Chinese LOSS was assessed by test–retest reliability. To that end, we randomly selected 300 nurses who volunteered to participate in the study. Two weeks after completing the questionnaire, the same 300 nurses were surveyed again via the same questionnaire. All reliability analyses were performed via Statistical Package for the Social Sciences (SPSS) version 25 software.

##### 2.3.3.3 Validity analysis

The validity analysis of the Chinese version of the LOSS involved two main aspects: content validity analysis and structural validity analysis. We used the Delphi method to assess content validity, which involved emailing experts. Eight renowned experts in the field of psychotherapy or psychological care from China were invited to rate each item on the scale individually, with ratings ranging from 1 (not relevant) to 4 (highly relevant). This scoring process was designed to determine the relevance of each item to the scale according to the scores provided by the experts. The item-level content validity index (I-CVI) and scale-level content validity index (S-CVI) were then calculated. When the number of experts was ≥ 6, the I-CVI was ≥ 0.78, and the S-CVI was ≥0.9, and therefore, the scale was considered to have good content validity (Weston et al., 2018). The structural validity analysis of the scale included exploratory factor analysis (EFA) and confirmatory factor analysis (CFA). SPSS version 25 software was used for EFA, and Analysis of Moment Structures (AMOS) version 24 software was used for CFA. We randomly divided the sample of 1,000 nurses into two groups: one (*n* = 500) group was analyzed via EFA, and the other (*n* = 500) was analyzed via CFA. First, Bartlett’s test of sphericity and the Kaiser–Meyer–Olkin (KMO) index were used to determine whether the data were suitable for factor analysis. When Bartlett’s test of sphericity was significant (*p* < 0.05) and the KMO value was >0.5, EFA could be performed. Principal Axis Factoring (PAF) and an oblique rotation method were used for EFA. The common factors with eigenvalues ≥1 were extracted, and the items with factor loadings < 0.4 and multiple loadings were deleted. Model fit was evaluated via CFA. A chi-squared test/degrees of freedom (*χ*^2^/DF) value <3, a root mean square error of approximation (RMSEA) value <0.08, and goodness-of-fit index (GFI), comparative fit index (CFI), and incremental fit index (IFI) values ≥0.9 indicated a good model fit and a stable scale structure (Weston et al., 2018).

## 3 Results

### 3.1 Results of the translation, back-translation, and transcultural adaptation of the LOSS

The experts agreed with most of the items on the LOSS. Owing to differences in cultural background, word choice, and sentence structure were only slightly inconsistent. Item 12 was changed from “People with emotional or financial problems are at greater risk of suicide” to “People with relationship problems or financial problems are at greater risk of suicide.” Item 16 was changed from “Very few people have suicidal thoughts” to “People who have suicidal thoughts are rare.” Item 25 was changed from “Only specialists can help those who want to die by suicide” to “Only psychological specialists can help those who want to commit suicide.” In the presurvey, each item was accepted and approved by 25 nurses and was considered easy to understand and unambiguous. The questionnaire could be completed within 5 min.

### 3.2 General population characteristics

A total of 1,000 nurses, including 35 males (3.5%) and 965 females (96.5%), from internal medicine, surgery, gynecology, pediatrics, intensive care, and oncology departments were included in this study. Among these nurses, 52.9% were in the age group between 30 and 39 years, 71.1% were married, and 99% had a bachelor’s degree or college diploma. In addition, 40.3% of the nurses were currently primary nurses. With respect to post placement, 90.6% of the nurses reported not holding a post. In addition, 64.7% of the nurses were employed on a contract basis. Further information on the general population is shown in Table 1.

**Table 1:** Frequency distribution of demographic characteristics (*n* = 1,000).

Factors	Group	*n*	%
Sex	Male	35	3.5
	Female	965	96.5
Age	<30	323	32.3
	30–39	529	52.9
	40–49	107	10.7
	≥50	41	4.1
Marital status	Unmarried	267	26.7
	Married	711	71.1
	Divorced	22	2.2
	Widowed	0	0
Education background	Doctor	0	0
	Master	6	0.6
	Bachelor or college degree	990	99
	Technical secondary school	4	0.4
Professional title	Nurse	170	17.0
	Primary nurse	403	40.3
	Nurse-in-charge	376	37.6
	Deputy director, nurse, and above	51	5.1
Post	Teaching teacher	10	1
	Nursing supervisor	75	7.5
	Head of the nursing department	4	0.4
	Nurse	906	90.6
	Else	5	0.5
Employment form	Contract system	647	64.7
	Personnel agency	111	11.1
	Authorized strength	106	10.6
	Else	136	13.6

### 3.3 Item analysis

The CR values of the 26 items of the Chinese version of the LOSS ranged from 15.093 to 41.768; all values were above 3, and the differences were statistically significant (*p* < 0.001 for all), indicating good discriminant validity for each item. These findings suggest that the Chinese version of the LOSS can be used to measure nurses’ suicide literacy effectively. The correlation coefficient between the scores of each item and the total scale score ranged from 0.450 to 0.712 (*p* < 0.001 for all), indicating that the homogeneity of each item and the scale was high. Therefore, all the items of the original scale were retained (Table 2).

**Table 2:** Item analysis for Chinese version of the LOSS.

Item	Critical ratio	Correlation coefficient between item and total score	Cronbach’s Alpha if item deleted
Signs of suicide-1	31.990	0.645	0.930
Signs of suicide-2	23.013	0.576	0.931
Signs of suicide-3	21.099	0.513	0.932
Signs of suicide-4	33.974	0.664	0.929
Signs of suicide-5	15.093	0.450	0.933
Risk factors for suicide-1	17.668	0.529	0.931
Risk factors for suicide-2	31.206	0.694	0.929
Risk factors for suicide-3	32.946	0.657	0.930
Risk factors for suicide-4	33.655	0.664	0.929
Risk factors for suicide-5	22.604	0.598	0.930
Risk factors for suicide-6	36.061	0.712	0.929
Risk factors for suicide-7	32.898	0.673	0.929
The cause or nature of suicide-1	22.188	0.592	0.930
The cause or nature of suicide-2	33.193	0.685	0.929
The cause or nature of suicide-3	27.734	0.643	0.930
The cause or nature of suicide-4	19.142	0.559	0.931
The cause or nature of suicide-5	23.586	0.622	0.930
The cause or nature of suicide-6	32.774	0.650	0.930
The cause or nature of suicide-7	25.831	0.629	0.930
The cause or nature of suicide-8	22.547	0.606	0.930
The cause or nature of suicide-9	21.504	0.552	0.931
The cause or nature of suicide-10	23.260	0.615	0.930
Treatment or prevention of suicide-1	26.102	0.596	0.930
Treatment or prevention of suicide-2	41.768	0.696	0.929
Treatment or prevention of suicide-3	25.571	0.471	0.930
Treatment or prevention of suicide-4	16.789	0.468	0.932

### 3.4 Reliability analysis

Cronbach’s *α* coefficient of the Chinese version of the LOSS was 0.933, Cronbach’s α coefficients of the four dimensions were 0.832, 0.893, 0.898, and 0.827, and the split-half reliability was 0.818. The split-half reliabilities of the four dimensions were 0.835, 0.877, 0.890, and 0.819, indicating that the scale had high internal consistency. Two weeks later, 300 nurses were surveyed again. Correlation analysis revealed that the test–retest reliability of the Chinese version of the LOSS was 0.889 (*p* < 0.001), and the test–retest reliabilities of the four dimensions were 0.890, 0.885, 0.892, and 0.904 (all *p* < 0.001). Therefore, the translated scale showed appropriate reliability. The specific indicators are shown in Table 3.

**Table 3:** Reliability analysis for Chinese version of the LOSS.

The scale and its dimension	Cronbach’s Alpha	Split-half reliability	Test–retest reliability
The LOSS	0.933	0.818	0.889
Signs of suicide	0.832	0.835	0.890
Risk factors for suicide	0.893	0.877	0.885
the cause or nature of suicide	0.898	0.890	0.892
treatment or prevention of suicide	0.827	0.819	0.904

### 3.5 Validity analysis

#### 3.5.1 Content validity analysis

Eight Chinese experts in psychotherapy or mental care assessed the content validity of the Chinese version of the LOSS. The results revealed that the I-CVI ranged from 0.875 to 1.000, and the S-CVI was 0.947, indicating that the Chinese version of the LOSS had good content validity.

#### 3.5.2 Exploratory factor analysis

The EFA results revealed that the KMO value was 0.946, and the result of Bartlett’s test of sphericity was statistically significant, indicating that EFA could be performed. Four factors with eigenvalues greater than 1, explaining 60.233% of the variance in the data, were extracted. In addition, the factor loading results were satisfactory. The specific indicators are shown in Table 4.

**Table 4:** Factor loadings of exploratory factor analysis for Chinese version of the LOSS.

Item	Factor 1	Factor 2	Factor3	Factor 4
Signs of suicide-1	0.956			
Signs of suicide-2	0.586			
Signs of suicide-3	0.418			
Signs of suicide-4	0.611			
Signs of suicide-5	0.560			
Risk factors for suicide-1		0.520		
Risk factors for suicide-2		0.866		
Risk factors for suicide-3		0.591		
Risk factors for suicide-4		0.577		
Risk factors for suicide-5		0.524		
Risk factors for suicide-6		0.862		
Risk factors for suicide-7		0.769		
The cause or nature of suicide-1			0.750	
The cause or nature of suicide-2			0.958	
The cause or nature of suicide-3			0.838	
The cause or nature of suicide-4			0.650	
The cause or nature of suicide-5			0.665	
The cause or nature of suicide-6			0.588	
The cause or nature of suicide-7			0.738	
The cause or nature of suicide-8			0.848	
The cause or nature of suicide-9			0.615	
The cause or nature of suicide-10			0.721	
Treatment or prevention of suicide-1				0.871
Treatment or prevention of suicide-2				0.859
Treatment or prevention of suicide-3				0.717
Treatment or prevention of suicide-4				0.770

#### 3.5.3 Confirmatory factor analysis

**Figure 1:**
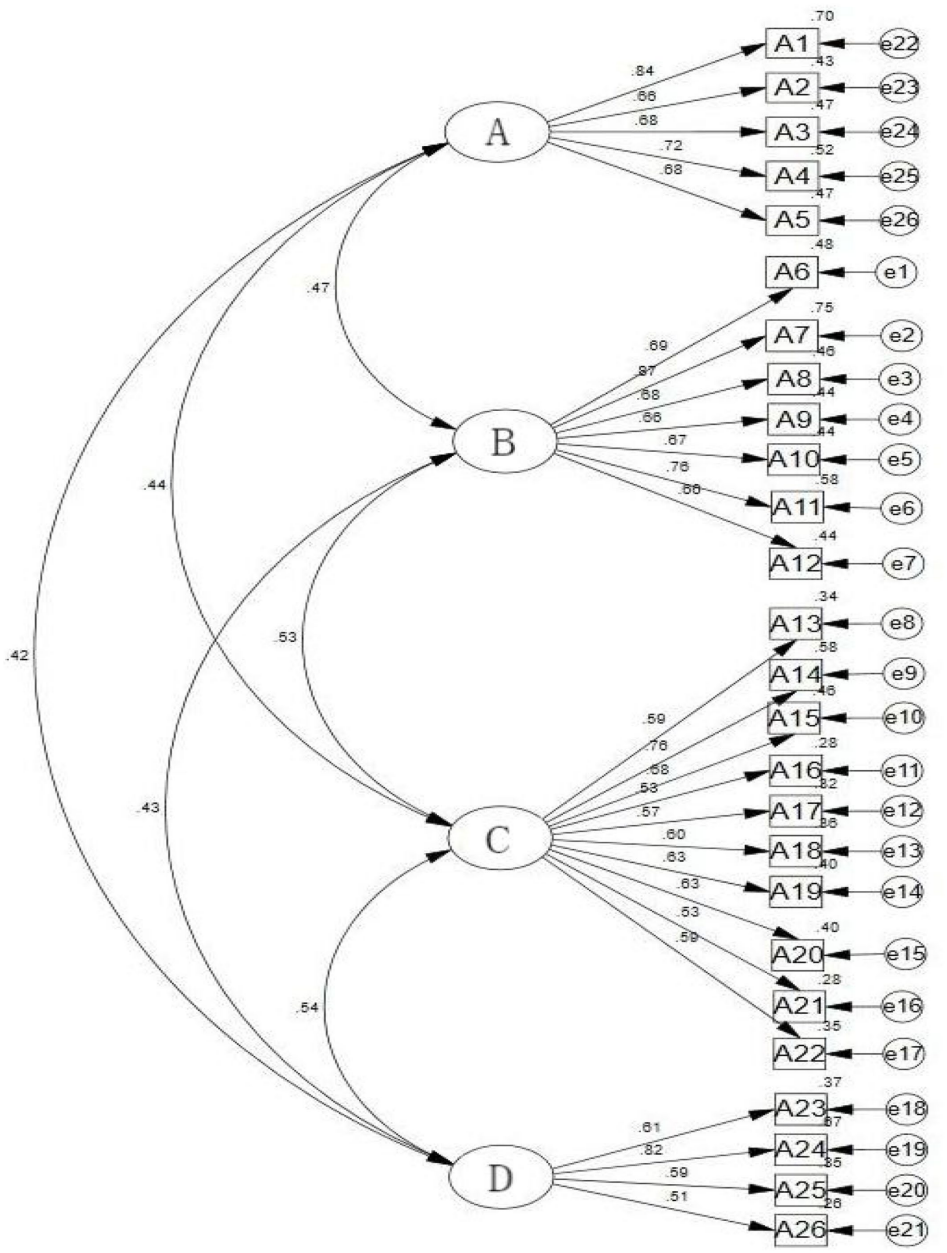
Standardized four-factor model of the Chinese version of the LOSS. **(A)** Signs of suicide; **(B)** risk factors for suicide; **(C)** the cause or nature of suicide; **(D)** the treatment or prevention of suicide.

The factor structure of the Chinese version of the LOSS was further verified via CFA. The results revealed that *χ*^2^/DF was 1.886, the RMSEA was 0.042, the GFI was 0.921, the CFI was 0.946, and the IFI was 0.947, indicating that the model had a good fit, as shown in Figure 1.

## 4 Discussion

Research has shown that nurses have lower suicide literacy (Karakaya et al., 2023). Therefore, early assessment of nurses’ suicide literacy level is crucial. Standardized assessments related to suicide include the Mental Health Literacy Scale (MHLS) and the Stigma of Suicide Scale (SOSS) (BinDhim et al., 2024; Ludwig et al., 2022). Although these scales have been successfully used for clinical healthcare workers (Clough et al., 2019; Wang et al., 2021), they still have limitations: the MHLS focuses on general mental health knowledge, lacks suicide-specific assessments, and does not include crisis intervention skills required by nurses. The SOSS mainly measures attitudes toward suicide victims rather than one’s own intervention ability, ignoring the hierarchical reporting system in China’s medical system. Compared with these standardized tools that have been successfully applied, the Literacy of Suicide Scale (LOSS) is constructed on the basis of the theory of mental health literacy (Batterham et al., 2013). The LOSS has a unique perspective, focusing more on the overall literacy level of individuals and covering various aspects such as signs of suicide, risk factors for suicide, the cause or nature of suicide, and the treatment or prevention of suicide. This comprehensive theoretical basis makes it more in line with the current concept of promoting holistic health and comprehensively improving mental health literacy in China; it also helps suicide prevention work from the root. The application of the LOSS in the nursing population presents three significant advantages. First, it meets professional needs. Nurses have daily contact with various types of patients, and this scale helps to evaluate nurses’ literacy regarding signs of suicide, risk factors for suicide, the reasons or nature of suicide, and the treatment or prevention of suicide; it also helps to better match the potential suicide risk response needs faced in nursing work. Second, it assists in precise intervention. This scale can help nurses have a clear understanding of their strengths and weaknesses in suicide prevention and other areas, thus improving their professional competence in a targeted manner and achieving more accurate identification of and intervention in patients’ suicide risk. Third, it can promote team collaboration. This scale is used for unified evaluation within the nursing community, facilitating team members in communicating and sharing experiences on suicide-related issues and enhancing the collaborative ability of the entire nursing team to respond to situations involving patient suicide. In addition, previous studies have shown that Western scales may have cultural response biases among Chinese nurses (Li et al., 2024), and the Chinese version of the LOSS has addressed this issue through cross-cultural adaptation.

The Chinese version of the LOSS consists of 26 items divided into four dimensions: signs of suicide, risk factors for suicide, the cause or nature of suicide, and the treatment or prevention of suicide. Its structure is the same as that of the original scale. Currently, the LOSS has been applied to nurses only in Türkiye, and it has not been applied to nurses in other countries (Karakaya et al., 2023). The translation process for the LOSS follows Brislin’s dual literal translation, back-translation model, which includes translation, back-translation, and expert consultation. After 8 Chinese experts were invited to revise the initial translation draft, the Chinese version of the LOSS was finally finalized. The preliminary survey results indicate that the Chinese version of the LOSS is clear and easy to understand.

The reliability and validity of the Chinese version of the LOSS were evaluated through an online questionnaire survey of 1,000 nurses in China. Reliability analysis checks whether a scale truly measures its structure (Koo and Li, 2016). In this study, Cronbach’s *α* coefficient of the Chinese version of the LOSS was 0.933, which was slightly greater than that of the original scale, indicating that the items in the Chinese version of the scale had greater internal consistency. Compared to attitude or skill scales, Cronbach’s α coefficient of knowledge-based scales is usually higher. The WHO Mental Health Literacy Study points out that Cronbach’s *α* values of knowledge scales are generally in the range of 0.85 ~ 0.95, which is related to the characteristics of measurement (Dey et al., 2020). Therefore, Cronbach’s α coefficient of the Chinese version of LOSS is relatively high, which is not a problem of item redundancy. Similarly, we found that the split-half reliability of the Chinese version of the LOSS was 0.818, and the split-half reliability of the four dimensions was 0.819–0.890, confirming the previous conclusions. Test–retest reliability refers to the consistency of results produced by repeated measurements of the same group of subjects via the same research tool, which reflects the stability of a test over time (Leppink and Pérez-Fuster, 2017). The test–retest reliability of the Chinese version of the LOSS was 0.889, and the test–retest reliability of the four dimensions ranged from 0.885 to 0.904, indicating that the Chinese version of the LOSS had good time stability. Overall, the Chinese version of the LOSS showed good reliability among Chinese nurses. Eight experts evaluated the content validity of the Chinese version of the questionnaire. The results revealed that the I-CVI values were between 0.875 and 1.000, and the S-CVI value was 0.947, better than the standard values of 0.780 and 0.900, respectively. Therefore, the scale has good content validity. The potential four-factor structure of the LOSS identified through EFA accounts for 60.233% of the total variance. In addition, the factor loadings for all 26 items are greater than 0.4. The factor structure of the translation scale is consistent with that of the original scale. Further validation of the potential factor structure was conducted using CFA, and all the fit indicators met the standard values. In summary, the Chinese version of the LOSS has good reliability and validity and is a reliable tool for evaluating the suicide literacy level of Chinese nurses. Therefore, this scale is suitable for widespread use and promotion in China.

In the Chinese healthcare system, nurses are among the groups that have the closest contact with hospitalized patients, and their level of suicide literacy directly affects the identification, intervention, and referral of high-risk suicide patients. The application of the Chinese version of the LOSS to the Chinese nursing community holds important theoretical and practical significance. Its theoretical significance includes verifying and expanding the applicability of the LOSS in the nursing population, verifying the applicability of the concept of “suicide literacy” in the Chinese nursing context, enriching the theoretical connotation of mental health literacy, and providing a theoretical basis for formulating “nursing patient” suicide prevention strategies that are in line with Chinese culture. At the same time, the theoretical positioning of nurses as “gatekeepers for suicide prevention among hospitalized patients” has been strengthened, and the theoretical dimension of nursing humanistic care has been expanded, thus promoting the cultural adaptability development of global nursing suicide prevention theory. As stable dimensions within the LOSS, the signs of suicide, risk factors for suicide, the cause or nature of suicide, and the treatment or prevention of suicide indicate cross-cultural consistency in the conceptualization of suicide literacy. The signs of suicide dimension emphasize that suicide is not a sudden event but, rather, an observable and gradual process. Nurses need to identify patients at high risk for suicide early in clinical practice, and the signs of suicide are directly related to nurses’ ability to observe and evaluate patients’ suicide risk. The characteristic of “enduring and expressing pain” in Chinese culture may make patients’ signs of suicide more covert, and nurses need to receive targeted training to identify early signs of patient suicide. The risk factors for suicide dimension indicate that the interaction of multiple factors causes suicide. In clinical practice, nurses may focus excessively on disease-related risks in hospitalized patients and overlook other nonmedical factors. Hospitals need to incorporate suicide risk assessment for hospitalized patients into routine nursing procedures to identify risk factors for patient suicide early. The cause or nature of suicide dimension involves the attribution of understanding of suicide. The traditional Chinese concept according to which ‘the body, hair, and skin are influenced by parents’ may reinforce nurses’ negative evaluation of hospitalized patients’ suicide, and hospitals need to adjust nurses’ cognition through training. The treatment or prevention of suicide dimension is used to evaluate the level of knowledge among nurses regarding the prevention and treatment of suicide in hospitalized patients. In clinical practice, nurses are not only executors but also responsible for health education, resource referrals, and other roles to prevent or treat suicide among hospitalized patients. The four dimensions of the Chinese version of the LOSS provide a structured theoretical framework for studying the suicide literacy level of Chinese nurses and can be used to evaluate the current situation and guide the optimization of intervention strategies. Subsequent qualitative research can be combined to reveal the cultural and social cognitive mechanisms underlying the various dimensions of the LOSS. From a practical perspective, the validated LOSS provides a standardized tool for evaluating the suicide literacy of Chinese nurses. The Chinese version of the LOSS can identify weak links in nurses’ knowledge related to suicide, thus encouraging hospitals to design precise continuing education courses (psychological first aid, communication skills, crisis intervention, etc.), promoting the inclusion of suicide prevention in standardized nursing training, and enhancing the overall response capabilities of the nursing team. Moreover, the Chinese version of the LOSS can be used to evaluate the differences in suicide literacy among nurses in different departments, hospitals, or regions. It provides a reference for health administrative departments to formulate mental health policies and encourages medical institutions to improve their suicide prevention systems.

In summary, this study tested the reliability and validity of the LOSS among Chinese nurses, which not only fills the gap in theoretical research on suicide literacy in the nursing field in China but also provides a scientific tool for clinical practice. Its value is reflected in the following: (1) at the theoretical level, it promotes knowledge innovation in nursing disciplines and facilitates interdisciplinary theoretical integration; (2) at the practical level, it optimizes nursing processes, improves medical quality, and ultimately serves patient safety and public health. In the future, we can conduct tests on suicide literacy scales for a more diverse population and further explore the measurement invariance of the scale across subgroups. At the same time, it is also possible to consider integrating the suicide literacy scale into the intervention research system, continuously expanding its theoretical depth and practical breadth.

### 4.1 Advantages and limitations

The revised Chinese version of the LOSS has high reliability and validity, and it is an effective tool for assessing the suicide literacy level of nurses in the Chinese context. However, we should also consider the limitations of this study. First, as this study involved only nurses from four Chinese provinces and municipalities, its findings may not be generalizable. Future research should test the scale in different cultural and population settings. Second, this study has methodological limitations due to its reliance on self-reported data, which may lead to social expectation bias and recall bias. To enhance the reliability of the results, it is recommended for future research to: (1) use a mixed method (such as behavioral observation + archival data) to implement triangulation; (2) reduce response bias through indirect questioning techniques; and (3) embedding social expectation measurement items into the scale for statistical correction. However, these methods require additional resource investment, and we need to balance the rigor and feasibility of our research. Finally, despite the successful validation of the psychometric properties of the Chinese version of the LOSS among nursing professionals, this study did not investigate potential correlates or predictors of suicide literacy. Therefore, investigating these factors will constitute a primary objective of our subsequent research endeavors.

## 5 Conclusion

The reliability and validity of the Chinese version of the LOSS were verified among Chinese nurses. This scale is a simple and reliable tool suitable for further promotion in China.

## Data Availability

The raw data supporting the conclusions of this article will be made available by the authors, without undue reservation.

## References

[ref2] Aldalaykeh M. Dalky H. Shahrour G. Rababa M. (2020). Psychometric properties of two Arabic suicide scales: stigma and literacy. Heliyon 6:e03877. doi: 10.1016/j.heliyon.2020.e03877, PMID: 32373752 PMC7193320

[ref3] Al-Shannaq Y. Aldalaykeh M. (2023). Suicide literacy, suicide stigma, and psychological help seeking attitudes among Arab youth. Curr. Psychol. 42, 6532–6544. doi: 10.1007/s12144-021-02007-9, PMID: 34177209 PMC8214717

[ref4] Bachmann S. (2018). Epidemiology of suicide and the psychiatric perspective. Int. J. Environ. Res. Public Health 15:425. doi: 10.3390/ijerph15071425, PMID: 29986446 PMC6068947

[ref5] Batterham P. J. Calear A. L. Christensen H. (2013). Correlates of suicide stigma and suicide literacy in the community. Suicide Life Threat. Behav. 43, 406–417. doi: 10.1111/sltb.12026, PMID: 23556504

[ref6] Bekaroğlu E. Bulut B. P. Demirbaş H. (2024). Reliability and validity of the suicide cognitions scale-revised (SCS-R) in emerging adulthood in Turkey. Death Stud. 48, 500–510. doi: 10.1080/07481187.2023.2240742, PMID: 37516976

[ref7] Bindhim N. F. Althumiri N. A. Ad-Dab'Bagh Y. Alqahtani M. Alshayea A. K. Al-Luhaidan S. M.. (2024). Exploring mental health literacy and its associated factors: a National Cross-Sectional Study in Saudi Arabia, 2023. Risk Manag Healthc Policy 17, 355–363. doi: 10.2147/RMHP.S442425, PMID: 38405268 PMC10893785

[ref8] Boukouvalas E. El-Den S. Murphy A. L. Salvador-Carulla L. O'Reilly C. L. (2020). Exploring health care Professionals' knowledge of, attitudes towards, and confidence in caring for people at risk of suicide: a systematic review. Arch. Suicide Res. 24, S1–S31. doi: 10.1080/13811118.2019.1586608, PMID: 30856366

[ref9] Calear A. L. Batterham P. J. Trias A. Christensen H. (2022). The literacy of suicide scale. Crisis 43, 385–390. doi: 10.1027/0227-5910/a000798, PMID: 34128704

[ref10] Chammas F. Januel D. Bouaziz N. (2022). Inpatient suicide in psychiatric settings: evaluation of current prevention measures. Front. Psych. 13:997974. doi: 10.3389/fpsyt.2022.997974, PMID: 36386981 PMC9650354

[ref11] Chan W. I. Batterham P. Christensen H. Galletly C. (2014). Suicide literacy, suicide stigma and help-seeking intentions in Australian medical students. Australas. Psychiatry 22, 132–139. doi: 10.1177/1039856214522528, PMID: 24526795

[ref12] Clough B. A. Ireland M. J. March S. (2019). Development of the SOSS-D: a scale to measure stigma of occupational stress and burnout in medical doctors. J. Ment. Health 28, 26–33. doi: 10.1080/09638237.2017.1370642, PMID: 28868957

[ref13] Dey M. Paz C. R. Jorm A. F. Marti L. Schaub M. P. Mackinnon A. (2020). Stigmatizing attitudes of Swiss youth towards peers with mental disorders. PLoS One 15:e235034. doi: 10.1371/journal.pone.0235034, PMID: 32706786 PMC7380889

[ref14] Fekih-Romdhane F. Amri A. Cheour M. (2022). Suicidal ideation, suicide literacy and stigma, disclosure expectations and attitudes toward help-seeking among university students: the impact of schizotypal personality traits. Early Interv. Psychiatry 16, 659–669. doi: 10.1111/eip.13211, PMID: 34477298

[ref15] Fontão M. C. Rodrigues J. Lino M. M. Lino M. M. Kempfer S. S. (2018). Nursing care to people admitted in emergency for attempted suicide. Rev. Bras. Enferm. 71, 2199–2205. doi: 10.1590/0034-7167-2017-0219, PMID: 30365784

[ref16] Gebhardt H. M. Ammerman B. A. Carter S. P. Stanley I. H. (2022). Understanding suicide: development and pilot evaluation of a single-session inpatient psychoeducation group. Psychol. Serv. 19, 423–430. doi: 10.1037/ser0000543, PMID: 35878069

[ref17] Gupta M. Esang M. Moll J. Gupta N. (2022). Inpatient suicide: epidemiology, risks, and evidence-based strategies. CNS Spectr. 28, 395–400. doi: 10.1017/S1092852922000918, PMID: 35860973

[ref18] Haddad M. Young N. (2022). Self-harm and suicide: occurrence, risk assessment and management for general nurses. Nurs. Stand. 37, 71–76. doi: 10.7748/ns.2022.e11911, PMID: 35502573

[ref19] Hagen J. Knizek B. L. Hjelmeland H. (2017). Mental health Nurses' experiences of caring for suicidal patients in psychiatric wards: an emotional endeavor. Arch. Psychiatr. Nurs. 31, 31–37. doi: 10.1016/j.apnu.2016.07.018, PMID: 28104055

[ref20] Han J. Batterham P. J. Calear A. L. Wu Y. Shou Y. van Spijker B. A. (2017). Translation and validation of the Chinese versions of the suicidal ideation attributes scale, stigma of suicide scale, and literacy of suicide scale. Death Stud. 41, 173–179. doi: 10.1080/07481187.2016.1214633, PMID: 27715477

[ref21] Henttonen P. Salmi J. Peräkylä A. Krusemark E. A. (2022). Grandiosity, vulnerability, and narcissistic fluctuation: examining reliability, measurement invariance, and construct validity of four brief narcissism measures. Front. Psychol. 13:993663. doi: 10.3389/fpsyg.2022.993663, PMID: 36300061 PMC9589046

[ref22] Huang M. Liu Y. Wang J. Mo L. Wang Y. Chen L.. (2019). High rates of depression anxiety and suicidal ideation among inpatients in general hospital in China. Int. J. Psychiatry Clin. Pract. 23, 99–105. doi: 10.1080/13651501.2018.1539179, PMID: 30762438

[ref23] Jafari A. Moshki M. Mokhtari A. M. Ghaffari A. Nejatian M. (2023). Title page: psychometric properties of literacy of suicide scale (LOSS) in iranian population: long form. BMC Public Health 23:608. doi: 10.1186/s12889-023-15528-8, PMID: 36997983 PMC10064757

[ref24] Jones P. S. Lee J. W. Phillips L. R. Zhang X. E. Jaceldo K. B. (2001). An adaptation of Brislin's translation model for cross-cultural research. Nurs. Res. 50, 300–304. doi: 10.1097/00006199-200109000-00008, PMID: 11570715

[ref25] Karakaya D. Özparlak A. Önder M. (2023). Suicide literacy in nurses: a cross-sectional study. J. Clin. Nurs. 32, 115–125. doi: 10.1111/jocn.16205, PMID: 34985161

[ref26] Koo T. K. Li M. Y. (2016). A guideline of selecting and reporting Intraclass correlation coefficients for reliability research. J. Chiropr. Med. 15, 155–163. doi: 10.1016/j.jcm.2016.02.012, PMID: 27330520 PMC4913118

[ref27] Leppink J. Pérez-Fuster P. (2017). We need more replication research - a case for test-retest reliability. Perspect. Med. Educ. 6, 158–164. doi: 10.1007/S40037-017-0347-Z, PMID: 28390030 PMC5466566

[ref28] Li Y. Liu Y. Wen J. (2024). Correction: knowledge, attitude, and practice toward value-based care among Chinese nurse: a cross-sectional study. BMC Nurs. 23:734. doi: 10.1186/s12912-024-02428-4, PMID: 39390509 PMC11465637

[ref29] Ludwig J. Dreier M. Liebherz S. Härter M. von Dem K. O. (2022). Suicide literacy and suicide stigma - results of a population survey from Germany. J. Ment. Health 31, 517–523. doi: 10.1080/09638237.2021.1875421, PMID: 33522335

[ref30] Muehlenkamp J. J. Grande N. Talbott M. (2023). Evidence-based vs informal suicide training: nurse confidence and comfort with suicidal patient care. J. Emerg. Nurs. 49, 266–274. doi: 10.1016/j.jen.2022.12.003, PMID: 36599734

[ref31] Sabrinskas R. Hamilton B. Daniel C. Oliffe J. (2022). Suicide by hanging: a scoping review. Int. J. Ment. Health Nurs. 31, 278–294. doi: 10.1111/inm.12956, PMID: 34825469

[ref32] Senf B. Maiwurm P. Fettel J. (2022). Attitudes and opinions towards suicidality in professionals working with oncology patients: results from an online survey. Support Care Cancer 30, 1775–1786. doi: 10.1007/s00520-021-06590-2, PMID: 34599381 PMC8727409

[ref33] Shao Q. Wang Y. Hou K. Zhao H. Sun X. (2021). The psychological experiences of nurses after inpatient suicide: a meta-synthesis of qualitative research studies. J. Adv. Nurs. 77, 4005–4016. doi: 10.1111/jan.14885, PMID: 34085728

[ref34] Shih J. Sellers C. M. Gavis-Hughson S. Fein A. O'Brien K. (2023). Experiences of inpatient psychiatric nursing care among people who have survived a suicide attempt. Issues Ment. Health Nurs. 44, 144–151. doi: 10.1080/01612840.2022.2164097, PMID: 36669129

[ref35] Snowdon J. Choi N. G. (2020). Undercounting of suicides: where suicide data lie hidden. Glob. Public Health 15, 1894–1901. doi: 10.1080/17441692.2020.1801789, PMID: 32744898

[ref36] Viard M. C. Grandgenèvre P. Bubrovszky M. Coisne E. Plancke L. Notredame C. E.. (2023). Impact of the suicidal crisis intervention training program on the confidence and skills of hospital professionals in the Hauts-de-France region. Encéphale 49, 504–509. doi: 10.1016/j.encep.2022.05.005, PMID: 35985851

[ref37] Wan Q. Ding X. Hu D. Han Y. Wang S. Liu Y.. (2020). A study of the epidemiology and risk factors for attempted suicide and suicide among non-psychiatric inpatients in 48 general hospitals in Hubei province, China, 2015-2017. Gen. Hosp. Psychiatry 63, 21–29. doi: 10.1016/j.genhosppsych.2019.06.003, PMID: 31230862

[ref38] Wang A. Jia S. Shi Z. Sun X. Zhu Y. Shen M. (2021). Validation and psychometric testing of the Chinese version of the mental health literacy scale among nurses. Front. Psychol. 12:791883. doi: 10.3389/fpsyg.2021.791883, PMID: 35153915 PMC8826253

[ref39] Warholak T. Barner J. C. Unni E. Thomas T. F. Devraj R. Quiñones-Boex A. C.. (2023). Reliability and validity evidence for an academic gender equity questionnaire. Am. J. Pharm. Educ. 87:ajpe9049. doi: 10.5688/ajpe9049, PMID: 36332918 PMC10159034

[ref40] Weston M. J. Verran J. A. Clavelle J. T. Porter-Oʼgrady T. (2018). Professional governance scale: instrument development and content validity testing. ANS Adv. Nurs. Sci. 41, 188–198. doi: 10.1097/ANS.0000000000000200, PMID: 29474228

[ref41] Yamaguchi S. Foo J. C. Sasaki T. (2023). A survey of suicide literacy in Japanese school teachers. Sci. Rep. 13:23047. doi: 10.1038/s41598-023-50339-2, PMID: 38155213 PMC10754903

[ref42] Zilinskas E. Lesinskienė S. (2023). Suicide literacy and attitudes toward psychological help-seeking: a cross-sectional study of students. J. Int. Med. Res. 51:3000605231172452. doi: 10.1177/03000605231172452, PMID: 37194200 PMC10192667

